# Effects of Dietary Supplementation with Thymol on Growth Performance, Blood Biochemistry, Rumen Fermentation Parameters, and Rumen Microbiota in Texel-Hazak Ewes

**DOI:** 10.3390/life16071162

**Published:** 2026-07-14

**Authors:** Shuhao Zong, Guoqiang Wei, Xingnuo Feng, Yekenxi Yeliubaidawa, Haifeng Deng, Hai Li, Yuhui Ma, Xiaobin Li, Xiang Luo, Sitong Chen, Kailibinuer Abulaiti, Zeen Chen

**Affiliations:** 1 College of Animal Science, Xinjiang Agricultural University, Urumqi 830052, China; zongshuhao418935@163.com (S.Z.); 15026032042@163.com (G.W.); 13239018728@163.com (X.F.); 13579816931@163.com (X.L.); 13391933918@163.com (S.C.); 17590555258@163.com (K.A.); 18261766361@yeah.net (Z.C.); 2 Yili Prefecture Zhaosu Horse Farm Agriculture and Animal Husbandry Development Co., Ltd., Yining 835600, China; 15719958980@163.com (Y.Y.); 15829601676@163.com (H.D.); 3 Zhaosu County Bureau of Agriculture and Rural Affairs, Yining 835600, China; 15739059024@163.com (H.L.); mayuhuim@126.com (Y.M.)

**Keywords:** thymol, replacement ewes, rumen fermentation, rumen microorganisms

## Abstract

This experiment aimed to study the effects of thymol on the growth performance, blood biochemical indices, hormone levels, rumen fermentation parameters, and rumen microbial changes of Texel-Hazak sheep ewes. Thirty healthy young hybrid ewes were selected and randomly divided into two groups, 15 each: a control group (CG) and an experimental group (EG). Under the same feeding management conditions, the diet of the CG group was not treated in any way, while the basal diet of the EG group was supplemented with 30 mg/kg of thymol. The experiment lasted 72 days, including a 12-day adaptation phase and a 60-day formal feeding period. During the formal feeding period, each ewe was offered 2 kg of diet per day on an as-fed basis. The thymol dose for the EG was calculated based on this planned daily feed allowance and was thoroughly mixed into the concentrate portion, which was completely consumed during individual feeding. The results showed that adding 30 mg/kg thymol to the diet significantly increased total weight gain and average daily gain in ewes (*p* < 0.01), and significantly decreased the feed-to-gain ratio (*p* < 0.01). It also significantly increased plasma P4 levels (*p* < 0.05) and significantly decreased B levels (*p* < 0.01). Meanwhile, the 16S rRNA sequencing results further confirmed that thymol altered ruminal microbial diversity and the relative abundance of specific bacterial taxa. These results suggest that the addition of 30 mg/kg thymol to the diet may act as a nutritional regulator, providing important insights into improving physiological status and rumen health during the growth stage of Texel-Hazak replacement ewes.

## 1. Introduction

Replacement ewes, as a backbone of the meat sheep industry chain, are not only the main source of the next generation of breeding ewes but also a key component in ensuring the continuity of meat sheep production, improving the genetic level of flocks, and maintaining economic benefits. The growth process of replacement ewes from physical maturity to sexual maturity is a critical stage in their reproductive life cycle, during which the growth performance and physiological condition of the ewes can have a profound impact on their subsequent reproductive performance. In recent years, with ongoing research into natural feed additives, thymol, a monoterpenoid phenol extracted from plants of the Lamiaceae family, has received widespread attention due to its broad-spectrum antibacterial, anti-inflammatory, antioxidant, and gut microbiota-modulating properties [1]. Studies on thymol as a feed additive have mainly focused on promoting animal growth performance and regulating the gut microbiota. Research has found that thymol can enhance the antioxidant capacity and immunity of broilers, improve intestinal morphology, and thereby increase growth performance [2]. Liu Zhonghao [3] found that adding thymol to the diet of weaned piglets can increase the relative abundance of Firmicutes and Bacteroidetes in the colon of weaned piglets, reduce the relative abundance of Escherichia coli, and decrease the incidence of diarrhea in piglets. At present, research on sheep farming activities mostly focuses on lamb growth, meat sheep fattening, and ewe pregnancy, while studies on replacement ewes from sexual maturity to physical maturity are scarce. Whether thymol can promote the growth and development of ewes by regulating hormone levels and rumen environment needs further investigation. Therefore, this experiment takes Texel-Hazak ewes as the research object, exploring whether adding thymol to the diet of ewes can affect the growth and development of replacement ewes by influencing their blood biochemical indicators, hormone levels, rumen fermentation parameters, and rumen microbial diversity, providing a reference for the scientific farming of replacement ewes.

## 2. Materials and Methods

### 2.1. Ethical Considerations

All animal care and experimental procedures adhered to the Guidelines for the Careand Use of Experimental Animals in China and were approved by the Experimental Animal Ethics Committee of the Institute of Xinjiang Agricultural University (Approval No. 2020032; Date of approval: 7 May 2020).

### 2.2. Experimental Materials

The thymol used in this study was purchased from Qingdao Mude Biotechnology Co., Ltd. (Qingdao, China), with a thymol content of ≥90%.

### 2.3. Experimental Design

The animal trial was conducted at a Suffolk sheep farm in Ili Hazakh Autonomous Prefecture, Xinjiang, China. Thirty healthy young third-generation Texel-Hazak crossbred ewes (hereinafter referred to as “Texel-Hazak ewes”) with similar age (13 ± 0.5 months) and body weight (43.65 ± 6.05 kg) were randomly assigned to two dietary treatment groups: the control group (CG) and the experimental group (EG), with 15 ewes per group. The CG was fed only the basal diet, while the EG added 30 mg/kg thymol to the feed based on the weight of the diet. The trial lasted for 72 days, including a 12-day adaptation phase and a 60-day formal feeding period. All ewes were raised under identical management and nutritional conditions, with the sole difference being the dietary supplementation strategy. Ewes in the CG and EG were housed separately according to dietary treatment in two pens located in the same sheep barn. Each pen contained 15 ewes, and the two pens were maintained under comparable environmental and management conditions, including similar space allowance, ventilation, bedding, cleaning frequency, feeding schedule, and free access to drinking water. The two treatment groups were not co-mingled during the trial. During the formal feeding period, each ewe was offered 2 kg of diet per day on an as-fed basis, divided into two equal feedings at 08:00 and 18:30. Mountain grass and alfalfa were used as roughage, whereas the remaining ingredients constituted the concentrate portion of the diet. For the EG, thymol was incorporated into the concentrate portion at a dose equivalent to 30 mg/kg of the planned daily diet allowance. At each feeding, ewes were temporarily separated using portable individual feeding pens. The concentrate portion was offered first and was generally consumed completely within approximately 10 min. The roughage portion was then provided, and the entire feeding process lasted approximately 30–40 min. Feed refusals were collected and weighed after feeding. Actual feed intake was calculated as the amount of feed offered minus feed refusals. Because the thymol-containing concentrate was completely consumed at each feeding, this feeding procedure ensured that each ewe in the EG received the intended daily thymol dose. This operation ensures that each ewes in the EG receives the same dose of thymol. The selected thymol doses were based on previous related studies [4]. The sheep pen was cleaned immediately after each feeding, and clean drinking water was available at all times. Throughout the trial period, the housing pens were regularly disinfected. The composition and nutritional levels of the basal diet are presented in Table 1.

### 2.4. Sample Collection and Processing

During the formal experimental period, the daily feed intake of the experimental ewes in each group was recorded. On days 0, 30, and 60 of the trial, before morning feeding, the ewes were driven to a floor scale to weigh and record their body weight. Average daily gain (ADG), total weight gain (TWG), and feed-to-gain ratio (F/G) were calculated. On the mornings of days 0, 30, and 60, before feeding and under fasting conditions, 10 mL of jugular venous blood was collected from each ewe into heparin sodium anticoagulant tubes. The blood was centrifuged at 3500 r/min for 15 min, and the plasma was collected and stored in 2 mL Eppendorf tubes at −20 °C for subsequent analysis of blood biochemical parameters and hormone indicators (using samples from day 30). On day 57 of the feeding trial, before morning feeding, rumen fluid was collected from each ewe using an oral stomach tube. To prevent contamination, the first 50 mL of rumen fluid drawn was discarded. Subsequently collected rumen fluid was filtered through four layers of gauze and aliquoted into pre-labeled sterile centrifuge tubes, then stored at −20 °C for subsequent determination of volatile fatty acids (VFA), ammonia nitrogen (NH_3_-N), and rumen microorganisms. The remaining rumen fluid samples were immediately analyzed on-site using a calibrated pH meter (FiveEasy22-Meter, Mettler Toledo International Trading (Shanghai) Co., Ltd., Shanghai, China) to measure rumen fluid pH.

### 2.5. Determination of Indicators and Methods

Blood biochemical parameters were measured using an automated biochemical analyzer. The tested items included: total protein (TP), albumin (ALB), globulin (GLB), creatinine (CREA), urea (UREA), glucose (Glu), total bilirubin (T-Bil), direct bilirubin (DBIL), triglycerides (TG), total cholesterol (TC), alanine aminotransferase (ALT), aspartate aminotransferase (AST), alkaline phosphatase (ALP), gamma-glutamyl transferase (γ-GT), creatine kinase (CK), and lactate dehydrogenase (LDH). The detection was performed using an automated biochemical analyzer (model BS-240VET, Mindray Bio-Medical Electronics Co., Ltd., Shenzhen, China) and the company’s commercial reagent kits, strictly following the manufacturer’s operating procedures. Plasma hormone indicators (including estradiol (E2), estrone (E1), dehydroepiandrosterone (DHEA), androstenedione (A4), aldosterone (Aldo), progesterone (P4), cortisol (F), and corticosterone (B) were measured using enzyme-linked immunosorbent assay (ELISA) kits. The assays were performed according to the kit’s accompanying instructions, and the content of the above plasma hormones was determined using a microplate reader (Agilent Technologies, Santa Clara, CA, USA, model: BioTek 800 TS). Specific operations followed the instructions provided with the detection kits from Nanjing Aoging Biotechnology Co., Ltd. (Nanjing, China).

The concentrations of total volatile fatty acids (TVFA), acetic acid, propionic acid, isobutyric acid, butyric acid, isovaleric acid, and valeric acid in the rumen fluid samples were determined using a high-performance liquid chromatography (HPLC) system (Waters-Baseline 520). The analysis conditions were as follows: wavelength 214 nm, mobile phase 0.02 mol/L KH_2_PO_4_/H_3_PO_4_ (pH = 2.37), flow rate 1 mL/min, C18 column temperature maintained at 30 °C, and injection volume 10 µL. The concentration of ammonia nitrogen (NH_3_-N) in the rumen fluid was determined using the phenol-hypochlorite colorimetric method.

According to the manufacturer’s instructions, total microbial genomic DNA was extracted from 20 rumen fluid samples using the E.Z.N.A.^®^ Soil DNA Kit (Omega Bio-tek, Norcross, GA, USA). DNA concentration and purity were determined using a NanoDrop 2000 spectrophotometer (Thermo Fisher Scientific, Wilmington, DE, USA), and DNA integrity was evaluated by 1% agarose gel electrophoresis. Qualified DNA samples were stored at −80 °C until further analysis. The V3–V4 hypervariable regions of the bacterial 16S rRNA gene were amplified using the universal primer pair 338F (5′-ACTCCTACGGGAGGCAGCAG-3′) and 806R (5′-GGACTACHVGGGTWTCTAAT-3′). PCR amplification was performed in a total reaction volume of 20 μL, containing 4 μL of 5× FastPfu Buffer, 2 μL of 2.5 mM dNTPs, 0.8 μL of forward primer (5 μM), 0.8 μL of reverse primer (5 μM), 0.4 μL of FastPfu DNA Polymerase (TransGen Biotech, Beijing, China), 10 ng of template DNA, and sterile deionized water to the final volume. The PCR program consisted of an initial denaturation at 95 °C for 3 min, followed by 27 cycles of denaturation at 95 °C for 30 s, annealing at 55 °C for 30 s, and extension at 72 °C for 45 s, with a final extension at 72 °C for 10 min. PCR products were verified by 2% agarose gel electrophoresis, purified using the AxyPrep DNA Gel Extraction Kit (Axygen Biosciences, Union City, CA, USA), and quantified using the QuantiFluor™-ST fluorescence quantification system (Promega, Madison, WI, USA). Purified amplicons were pooled at equimolar concentrations to construct sequencing libraries and subsequently subjected to paired-end sequencing (2 × 300 bp) on the Illumina MiSeq platform (Illumina, San Diego, CA, USA). Sequencing was performed by Majorbio Bio-Pharm Technology Co., Ltd. (Shanghai, China).

Raw sequencing reads were first demultiplexed according to sample-specific barcodes and quality-filtered using fastp software (version 0.23.4) to remove adapter sequences and low-quality reads. The processed sequences were subsequently analyzed in the QIIME2 platform (version 2024) using the DADA2 plugin (version 2024) for quality filtering, denoising, chimera removal, and paired-end read merging, thereby generating high-resolution amplicon sequence variants (ASVs). Compared with conventional operational taxonomic unit (ASV)-based clustering approaches, the ASV-based method provides higher taxonomic resolution at the single-nucleotide level. Representative ASV sequences were taxonomically assigned using a Naïve Bayes classifier against the SILVA 16S rRNA database (release 138). To minimize the influence of sequencing depth variation on microbial diversity analyses, all samples were rarefied to a standardized sequencing depth prior to downstream analyses. Alpha diversity indices, including Chao, ACE, Shannon, and Simpson indices, were calculated in QIIME2 to evaluate microbial richness and diversity within samples. Beta diversity was assessed based on Unweighted unifrac distance matrices, and principal coordinates analysis (PCoA) was performed to visualize differences in microbial community structures among groups. Linear discriminant analysis effect size (LEfSe) analysis (LDA > 4) was further conducted to identify significantly enriched microbial taxa among different groups. Functional prediction was performed using Tax4Fun2 (version 1.1.5) based on SILVA138 annotations. Predicted KEGG pathways were compared using Welch’s *t*-test. To control for false-positive results arising from multiple comparisons, false discovery rate (FDR) correction was performed using the Benjamini–Hochberg method, and adjusted *p*-values (q-values) < 0.05 were considered statistically significant where applicable. All statistical analyses and data visualizations were performed in the R software environment (version 4.3.1), and figures were generated using the ggplot2 package (version 3.5.1).

Correlation analyses between microbial taxa and phenotypic parameters, including growth performance, rumen fermentation characteristics, and plasma biochemical indices, were performed using Spearman’s rank correlation coefficient in GraphPad Prism software (version 10.0.0; GraphPad Software Inc., San Diego, CA, USA). Correlation coefficients (r) and corresponding *p*-values were calculated to evaluate the strength and significance of associations. Correlations with *p* < 0.05 were considered statistically significant. Correlation heatmaps were generated to visualize the relationships between microbial taxa and host phenotypic variables.

In this study, growth performance and blood biochemical parameters were collected from all 30 experimental ewes (*n* = 15 per group). Considering experimental feasibility and testing cost constraints, after the trial concluded and prior to data analysis, 10 ewes per group were randomly selected for hormone assays, rumen fermentation parameter analysis, and rumen microbial sequencing (*n* = 10 per group). The same 10 animals per group were used across hormone, rumen fermentation, and microbial sequencing analyses. No animals died or showed health abnormalities during the trial, and no samples were excluded. This sample size was determined based on the common practice in rumen microbiome studies of balancing analytical robustness with sequencing cost.

### 2.6. Data Processing and Statistical Analysis

All data are presented as mean ± standard error of the mean (SEM). Statistical analysis was performed using SPSS 27.0 software, with the significance level set at *p* < 0.05. The normality of distribution and homogeneity of variances of the data were first assessed. For data measured at multiple time points (e.g., plasma biochemical parameters), a repeated measures analysis of variance (ANOVA) was used, with “Treatment Group (TRT)” as the between-subjects factor and “Sampling Time” as the within-subjects factor. For data measured only at the end of the trial, a one-way ANOVA was used for inter-group comparisons. When the assumptions for parametric tests were not met, non-parametric tests were employed: the Friedman test for repeated measures data and the Kruskal-Wallis test for independent sample data. Post-hoc multiple comparisons following parametric tests were conducted using Duncan’s method.

The individual ewe was considered the experimental unit for growth performance, plasma biochemical indices, steroid hormone concentrations, rumen fermentation parameters, and rumen microbiota analysis. Thymol was incorporated into the diet of the experimental group at 30 mg/kg, and sample collection and data recording were performed at the individual-animal level.

## 3. Results

### 3.1. Dietary Supplementation of Thymol Affects the Growth and Development of Ewes

The data on the effect of dietary thymol supplementation on the growth and development of Texel-Hazak ewes are shown in Table 2 (Table S1). As seen in Table 2, compared to the CG, the final weight of the EG was significantly higher (*p* < 0.05), while the TWG and ADG were extremely significantly higher (*p* < 0.01), and the F/G was extremely significantly lower (*p* < 0.01). There were no significant differences in initial weight and actual feed intake between the CG and the EG (*p* > 0.05).

### 3.2. The Effect of Dietary Thymol Supplementation on Plasma Biochemical Indices in Ewes

The effects of dietary thymol supplementation on plasma biochemical parameters in ewes were evaluated using repeated-measures ANOVA, with results summarized in Table 3 (Table S2). The main treatment effect analysis revealed that dietary thymol supplementation significantly altered several plasma indicators. Compared with the CG group, the EG group exhibited significantly higher concentrations of TP, ALB, GLU, TC, and TG (*p* < 0.01), whereas D-Bil concentration was significantly lower (*p* < 0.05). The concentrations of LDL-C and AST and γ-GT were significantly reduced (*p* < 0.01). Time-related effects were also evident: sampling time significantly influenced ALB concentration, GLU concentration, and ALP activity (*p* < 0.05), and exerted highly significant effects on the concentrations of CREA, UREA, T-Bil, D-Bil, TC, TG, and HDL-C, as well as on AST, CK, and LDH activities, and the AST/ALT ratio (*p* < 0.01). Furthermore, significant treatment-by-time interactions were observed for D-Bil concentration, AST, and ALT activities (*p* < 0.05), and highly significant interactions for the concentrations of TP, ALB, GLB, CREA, GLU, TC, and TG, and for ALP and LDH activities (*p* < 0.01).

### 3.3. Effect of Dietary Thymol Supplementation on Steroid Hormone Levels in Ewes

**Figure 1:**
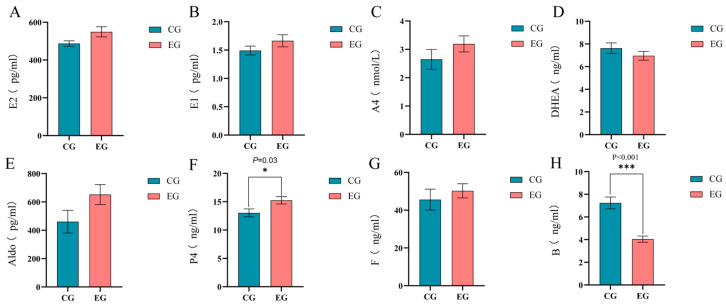
Effect of thymol on blood steroid hormone levels in ewes. (**A**) Estradiol index. (**B**) Estrone index. (**C**) Androstenedione index. (**D**) Dehydroepiandrosterone index. (**E**) Aldosterone index. (**F**) Progesterone index. (**G**) Cortisol index. (**H**) Corticosterone index. *n* = 10 per group, * *p* < 0.05, *** *p* < 0.001.

The data on the effect of dietary thymol supplementation on steroid hormone levels in Texel-Hazak ewes are shown in Figure 1 (Table S3). From Figure 1, it can be seen that compared with the CG, the plasma P4 concentration in the EG was significantly increased (*p* < 0.05), and the B concentration was significantly decreased (*p* < 0.01). There were no significant differences in the concentrations of E2, E1, A4, DHEA, Aldo, and F between the CG and the EG.

### 3.4. Effect of Dietary Thymol Supplementation on Rumen Fermentation Parameters in Ewes

**Figure 2:**
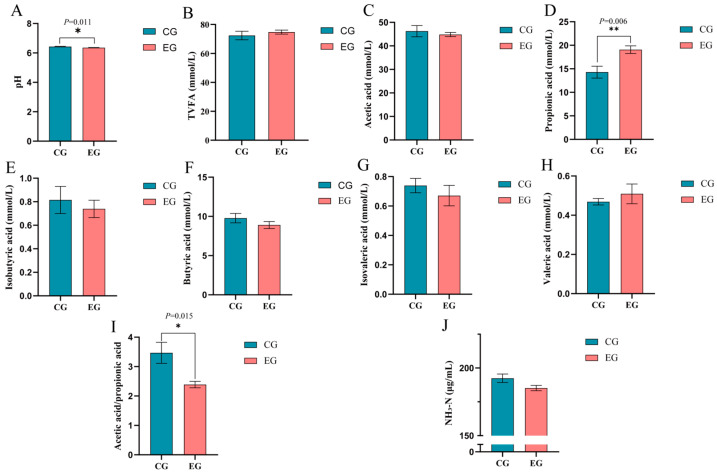
Effect of thymol on rumen fermentation parameters in ewes. (**A**) pH index. (**B**) TVFA index. (**C**) Acetic acid index. (**D**) Propionic acid index. (**E**) Isbutyric acid index. (**F**) Butyric acid index. (**G**) Isovaleric acid index. (**H**) Valeric acid index. (**I**) Acetic acid/propionic acid index. (**J**) NH_3_-N index. *n* = 10 per group, * *p* < 0.05, ** *p* < 0.01.

The data on the effect of dietary thymol supplementation on rumen fermentation parameters in Texel-Hazak ewes are shown in Figure 2 (Table S4). From Figure 2, it can be seen that compared with the CG, the pH in the EG was significantly decreased (*p* < 0.05), the propionic acid concentration was significantly increased (*p* < 0.01), and the acetic acid/propionic acid ratio was significantly decreased (*p* < 0.05). There were no significant differences in the concentrations of TVFA, acetic acid, isobutyric acid, butyric acid, isovaleric acid, valeric acid, and NH_3_-N between the CG and the EG (*p* > 0.05).

### 3.5. Effect of Dietary Thymol Supplementation on Rumen Microbial Diversity in Ewes

**Figure 3:**
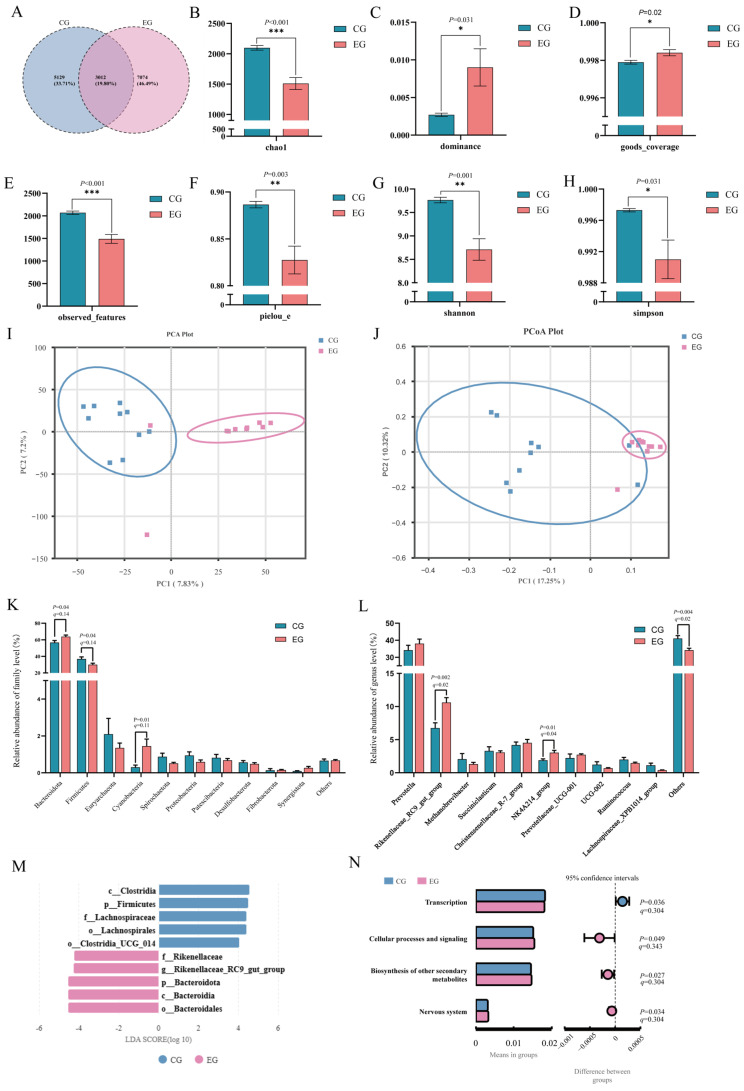
Effect of thymol on rumen microbial diversity in ewes. (**A**) Venn diagram analysis based on ASVs. (**B**) Chao1 Index. (**C**) Dominance index. (**D**) coverage Index. (**E**) Observed_features index. (**F**) Pielou_e index. (**G**) Shannon index. (**H**) Simpson index. (**I**) PCA analysis based on Euclidean distances. (**J**) PCoA analysis based on Unweighted unifrac distance. (**K**) Analysis of the relative abundance of the K-phylum level microbiota. (**L**) Analysis of the relative abundance of the horizontal microbiota of genus. (**M**) Differential microbial taxa between groups identified by linear discriminant analysis effect size (LEfSe) method (LDA score > 4.0, *p* < 0.05). (**N**) Microbial functional pathways with significantly different predicted abundances (*p* < 0.05) between CG and EG identified based on Tax4Fun prediction and *T*-test (the bar chart on the left shows the mean abundance of each pathway within groups, and the forest plot on the right displays the inter-group difference value and its 95% confidence interval). Significant correlations are marked with asterisks: * *p* < 0.05, ** *p* < 0.01, *** *p* < 0.001. For figures (**K**,**L**,**N**), raw *p*-values and FDR-adjusted *q*-values are shown. *q*-values were calculated using the Benjamini–Hochberg false discovery rate correction. Statistical significance was determined based on *q* < 0.05, whereas *q* ≥ 0.05 was considered not statistically significant. Comparisons with raw *p* < 0.05 but *q* ≥ 0.05 were considered nominal differences rather than statistically significant differences. *n* = 10 per group.

A total of 15,215 ASVs were detected in all rumen fluid samples. Among them, 3012 ASVs were shared by the CG and the EG, 5129 ASVs were unique to the CG, and 7074 ASVs were unique to the EG (Figure 3A). Based on alpha diversity analysis, compared with the CG, the Chao1 index, observed_features index, pielou_e index, and Shannon index in the EG were significantly increased (*p* < 0.01), while the dominance index and goods_coverage index were significantly decreased (*p* < 0.05), and the Simpson index was significantly increased (*p* < 0.05) (Figure 3B–H) (Table S5). The results of beta diversity analysis showed (Figure 3I) that based on PCA analysis using Euclidean distances, the samples from the CG and the EG were far apart, indicating large differences in community composition. Based on PCoA analysis using Unweighted UniFrac distance (Figure 3J), the samples from the CG and the EG were relatively far apart, indicating significant differences in microbial communities.

Analysis at the phylum level showed that Bacteroidota and Firmicutes were the dominant phyla (Figure 3K) (Table S5). Bacteroidota showed a nominal increase in the EG compared with the CG based on the raw *p*-value (*p* < 0.05), but this difference did not remain significant after FDR correction (*q* = 0.143). Firmicutes showed a nominal decrease in the EG based on the raw *p*-value (*p* < 0.05), but the difference was not significant after FDR correction (*q* = 0.143). Cyanobacteria also showed a nominal increase in the EG based on the raw *p*-value (*p* < 0.05), but this difference did not remain significant after FDR correction (*q* = 0.110). Analysis at the genus level (Figure 3L) (Table S5) showed that Prevotella and Rikenellaceae_RC9_gut_group were the dominant genera. After FDR correction, the relative abundance of Rikenellaceae_RC9_gut_group was significantly higher in the EG than in the CG (*p* < 0.01, *q* = 0.02). Similarly, NK4A214_group was significantly enriched in the EG compared with the CG (*p* < 0.01, *q* = 0.04). The relative abundance of the “Others” category was significantly lower in the EG than in the CG (*p* < 0.01, *q* = 0.02). No significant differences were observed for the other dominant genera after FDR correction (*q* > 0.05).

To further identify differences in rumen microbial communities between groups, LEfSe analysis was used to identify species with statistically significant differences at different taxonomic levels. As shown in Figure 3M, a total of 10 significantly different species were identified between the CG and the EG. Among them, the CG exhibited 5 significantly different species: Clostridia, Firmicutes, Lachnospiraceae, Lachnospirales, and Clostridia_UCG_014. The EG exhibited 5 significantly different species: Rikenellaceae, *Rikenellaceae_RC9_gut_group*, Bacteroidota, *Bacteroidia*, and Bacteroidales. Further predicted functional analysis showed that four functional pathways, including transcription, cellular processes and signaling, biosynthesis of other secondary metabolites, and nervous system, showed nominal differences between the CG and EG based on raw *p*-values (Figure 3N). However, these differences did not remain significant after FDR correction (*q* > 0.05). Therefore, the predicted functional differences should be interpreted as exploratory results rather than statistically significant functional alterations.

### 3.6. Correlation Analysis

**Figure 4:**
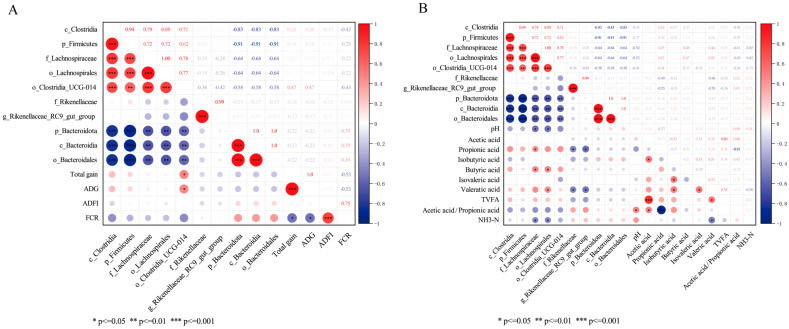
Correlation analysis: (**A**) Correlation analysis of growth performance and differential bacteria. (**B**) Correlation analysis of rumen fermentation parameters and differential bacteria.

To assess the potential connections among differential bacteria, growth performance and rumen fermentation parameters, we conducted a correlation analysis. The results of the correlation analysis between differential bacteria and growth performance showed that the differential bacteria o_Clostridia_UCG-014 was significantly positively correlated with TWG and ADG (Figure 4A). The results of the correlation analysis between differential bacteria and rumen fermentation parameters indicated that c_Clostridia and o_Clostridia_UCG-014 were significantly positively correlated with total volatile fatty acid concentration; f_Lachnospiraceae was significantly positively correlated with propionate, butyrate and TVFA concentration, and significantly negatively correlated with pH and NH_3_-N concentration; o_Lachnospirales was significantly negatively correlated with pH and NH_3_-N concentration, and significantly positively correlated with butyric acid, valeric acid and TVFA; f_Rikenellaceae was significantly negatively correlated with propionic acid and valeric acid concentration; g_Rikenellaceae_RC9_gut_group was significantly negatively correlated with propionic acid, valeric acid and TVFA (Figure 4B).

## 4. Discussion

### 4.1. Dietary Supplementation of Thymol Affects the Growth and Development of Ewes

Body weight is an important indicator in the growth and development of animals, reflecting growth rate, growth status, feeding management, nutritional level of feed, and the physical characteristics of the animals. In production practices, body weight is also used to evaluate different stages such as sexual maturity, suitability for first mating, and physical maturity. For example, in large herbivorous animals like cows, 55–60% of adult body weight is usually taken as the standard for entering the breeding stage; for ewes, 65–70% [5] of adult body weight is generally considered suitable for entering the breeding stage. Therefore, changes in body weight are not only an important indicator of growth and development in young sheep but also a critical criterion for evaluating sexual and physical maturity. This study found that by the end of the experiment, the body weights of the two groups of hybrid ewes reached 54.78 kg and 57.19 kg, exceeding the suitable first mating weight of 45 kg for ewes. Thymol, a monoterpene phenol extracted from plants, is widely used in animal production due to its broad-spectrum antibacterial, antioxidant, and other physiological functions. Studies have found that thymol can improve the ileal morphological structure and enhance intestinal barrier function, thereby promoting the growth and development of broilers [6]. Wang Xinyuan et al. [7] found that dietary supplementation of oregano essential oil could improve the growth performance of growing pigs by increasing the apparent digestibility of nutrients. In this trial, by adding 30 mg/kg of thymol to the ewes’ diet, the TWG and ADG of the EG were significantly higher than those of the CG. This result is consistent with previous studies, indicating that thymol has a positive effect on promoting the growth and development of ewes. In the present study, dietary thymol supplementation significantly increased TWG and ADG and decreased the F/G in replacement ewes. The lower F/G was mainly associated with the higher body-weight gain observed in the EG under the present feeding conditions.

### 4.2. The Effect of Dietary Thymol Supplementation on Plasma Biochemical Indices in Ewes

Plasma biochemical indicators are important parameters reflecting the material metabolism and physiological functions of animals [8]. Plasma TP content is a key indicator of protein metabolism, with ALB, as the main protein synthesized by the liver, playing a vital physiological role in maintaining plasma colloid osmotic pressure and substance transport. GLB are primarily involved in the immune response and are crucial for maintaining immune function. Studies have shown that the combined use of thymol and calcium propionate can increase plasma ALB levels in broilers aged 1–21 days [9]. In this trial, the addition of thymol to the ewes’ diet significantly increased the concentrations of TP and ALB in the blood. Furthermore, the liver converts amino acids, plasma proteins, and ammonia produced from protein metabolism into UREA. In this trial, UREA levels in the EG showed an increasing trend compared to the CG. As the central organ for lipid synthesis and transport, the liver not only synthesizes lipids such as cholesterol (Chol) and TG but also produces and degrades lipoproteins, responsible for lipid transport in the blood. Thymol increased plasma Chol and TG levels in ewes while reducing LDL-C content. LDL-C, the primary lipoprotein for Chol transport in the blood, is both synthesized and catabolized in the liver [10]. Additionally, TG and Glu are indicators reflecting the energy metabolism status of animals; insufficient energy intake leads to decreased plasma levels of TG and Glu. In this trial, TG and Glu levels were significantly higher in the thymol group. Therefore, dietary supplementation of thymol in ewes has a promoting effect on hepatic protein metabolism, lipid metabolism, and energy metabolism. plasma AST and ALT levels are important indicators for evaluating liver damage, with their elevation often accompanying metabolic disorders in the liver [11]. D-Bil is formed from I-Bil through conjugation with glucuronic acid in the liver and is primarily excreted via bile, influenced by bile duct patency [12]. Similarly, γ-GT transferase is a key indicator for assessing liver and biliary function, and its increased plasma levels are typically associated with liver damage and biliary obstruction [13]. As a plant extract, thymol possesses excellent antioxidant properties. Previous studies have found that thymol can significantly reduce the elevated levels of ALT, AST, and bilirubin in plasma in acetaminophen-induced hepatotoxicity models in rats, thereby protecting the liver. In this trial, thymol reduced plasma levels of AST, D-Bil, and γ-GT in ewes, suggesting that thymol may have potential health-related implications for hepatic function in ewes.

### 4.3. Effect of Dietary Thymol Supplementation on Steroid Hormone Levels in Ewes

Steroid hormones play important roles in regulating endocrine homeostasis and reproductive physiology in mammals. P4 is an important steroid hormone involved in endocrine regulation and reproductive function [14,15]. In the present study, dietary thymol supplementation was associated with increased circulating P4 concentrations in replacement ewes, suggesting that thymol may be related to changes in steroid hormone profiles. However, because estrous-cycle stage was neither controlled nor recorded at the time of sampling, the hormone results should be interpreted only as steroid hormone profiles under the present sampling conditions. In addition, reproductive traits were not evaluated in the current study; therefore, the biological significance and underlying mechanisms of this hormonal response remain unclear and require further investigation. plasma B levels are commonly used to assess stress levels in animals. Cold stress can elevate B hormone levels, leading to increased oxidative stress in oocytes, a reduction in the number of mature oocytes, and impaired reproductive performance [16,17]. As an antioxidant, thymol can increase the redness value and glutathione peroxidase activity in the breast muscle of heat-stressed broilers, reduce muscle lipid peroxidation, and thereby enhance muscle oxidative stability. In the present study, thymol reduced plasma B concentration in ewes, suggesting that thymol may have potential value in alleviating stress responses in ewes. However, because antioxidant indices were not measured in this study, the mechanisms underlying its potential stress-alleviating effects require further verification.

### 4.4. Effect of Dietary Thymol Supplementation on Rumen Fermentation Parameters in Ewes

Maintaining the homeostasis of the ruminal environment is the physiological basis for the health and production performance of ruminants. Among various parameters, ruminal pH is a key indicator for assessing fermentation status and regulating the degradation and absorption of nutrients. It not only sensitively reflects internal environmental stability but is also crucial for maintaining normal rumen function [18]. In this trial, the rumen fluid pH of ewes in the CG and the EG was 6.47 and 6.39, respectively, both falling within the normal physiological range (6–7) [19]. Thymol significantly increased the propionate content in the rumen fluid of ewes. Volatile fatty acids (VFAs) are the primary factors affecting ruminal pH, and a statistically negative correlation exists between VFA levels and pH. In this trial, although the total VFA content showed no significant change, a significant difference in pH was still observed, which may be related to variations in lactate content in the rumen [20]. Studies have found that compared to the phylum Firmicutes, the phylum Bacteroidetes contains a larger proportion of genes encoding cellulolytic glycoside hydrolases and a greater number of carbohydrate-active enzyme gene clusters, endowing Bacteroidetes with superior cellulose-degrading capabilities [21]. Meanwhile, within Bacteroidetes, the genus Prevotella, as an important starch-degrading bacterium, can produce propionate as a key fermentation product. In this trial, among the dominant phyla Bacteroidetes and Firmicutes, the relative abundance of Bacteroidetes significantly increased, while that of Firmicutes significantly decreased. This shift is likely the main reason for the elevated propionate concentration in the rumen. Research indicates that increased feed intake, a higher proportion of non-fiber carbohydrates, and reduced forage particle length can all enhance the concentration of substrates available for microbial fermentation per unit time from dietary degradation in the rumen, thereby lowering the acetate-to-propionate ratio [22]. However, this trial did not involve changes in feed composition, and the F/G decreased in the thymol-supplemented group. These findings suggest that the decreased ruminal acetate-to-propionate ratio in ewes may be associated with changes in ruminal microbial community structure. NH3-N, as a product of protein degradation, not only provides the primary nitrogen source for microbial protein synthesis but also regulates the activity of fiber- and starch-degrading bacteria. It is a key biomarker reflecting nitrogen metabolism balance in the rumen, and its dynamic changes directly reflect the efficiency of microbial conversion of dietary protein. In this trial, dietary supplementation with thymol showed a trend of increasing the concentration of ammonia nitrogen in the rumen. This may be attributed to thymol’s regulation of protein-degrading bacteria such as the genus Prevotella and the phylum Synergistota [23,24].

### 4.5. Effect of Dietary Thymol Supplementation on Rumen Microbial Diversity in Ewes

The intestine is an important and complex internal environment in animals, and the microorganisms within the gut are the primary factor constituting this environment. The gut harbors a vast diversity of microbial species with intricate functions. Together with intestinal contents and tissues, they form a complex, dynamic, and homeostatic relationship that collectively regulates the digestion and absorption of nutrients in the animal’s intestine, playing a crucial role in intestinal health and the growth and development of the animal. Thymol, a monoterpene phenol extracted from plants of the genus Thymus, can modulate the gut microbiota and improve microbial balance. Studies have found that thymol can enhance rumen fermentation and reduce methane production by increasing the relative abundance of Firmicutes and decreasing that of Bacteroidetes in the rumen [25]. Yuan et al. [26] discovered that dietary supplementation with thymol increased the relative abundance of Bifidobacterium, Fusobacterium, and Allobaculum in the intestines of blue foxes, improved intestinal immune barrier function, and promoted their growth and development.

Based on Venn diagram analysis, microbial alpha diversity, and beta diversity analyses, this study revealed significant differences between the CG and the EG. This indicates that dietary supplementation with thymol significantly influenced the community structure of the ruminal microbiota in ewes. Analysis at the phylum level showed that thymol increased the relative abundance of Bacteroidetes and Cyanobacteria in the rumen while decreasing that of Firmicutes. As the two dominant phyla in the rumen of ewes, Firmicutes and Bacteroidetes can affect host nutrient digestion, absorption, and intestinal immune homeostasis through their metabolites and immunomodulatory mechanisms. Some bacterial taxa within Firmicutes, including members of Ruminococcaceae and Lachnospiraceae, have been associated with plant structural polysaccharide fermentation and short-chain fatty acid production, such as acetate and butyrate [27]. In addition, certain taxa within Bacteroidetes, especially Prevotella-related bacteria, have been linked to the utilization of dietary polysaccharides and proteins and to propionate production in the rumen [28]. Additionally, the Firmicutes-to-Bacteroidetes ratio (F/B) is commonly used to assess gut microbiota status. In the present study, the addition of thymol to the diet decreased the F/B ratio in the rumen of ewes, indicating a shift in the phylum-level structure of the ruminal microbial community. This change occurred together with increased ruminal propionate concentration and a decreased acetate-to-propionate ratio, suggesting that the altered F/B ratio may be associated with changes in ruminal fermentation pattern. Because Firmicutes and Bacteroidetes are broad bacterial phyla comprising diverse taxa with different ecological and metabolic functions, the observed changes in their relative abundance should be regarded as taxonomic shifts in the ruminal microbial community rather than being interpreted simply as beneficial or detrimental, and their functional significance requires further interpretation at lower taxonomic levels.

Analysis at the genus level revealed that thymol increased the relative abundance of *Rikenellaceae_RC9_gut_group* and *NK4A214_group* in the rumen while decreasing the relative abundance of other genera. Studies have found that gradually replacing rumen contents with rice straw in an in vitro rumen fermentation system led to an increase in the relative abundance of *Rikenellaceae_RC9_gut_group*, making it one of the dominant genera in the later stages of fermentation [29]. Other research indicates that *Rikenellaceae_RC9_gut_group* can produce ethanol, acetate, and butyrate by degrading lignocellulose [30]. *NK4A214_group* belongs to the family Ruminococcaceae and can degrade plant cell walls by producing cellulases, thereby promoting the nutrient absorption of high-fiber feed in ruminants [31]. Furthermore, *NK4A214_group* shows a significant positive correlation with propionate and isovalerate [32]. In this trial, both the relative abundance of *NK4A214_group* and propionate content significantly increased. Therefore, these findings suggest that thymol may influence the ruminal fermentation environment by modulating the microbial community structure.

LEfSe analysis results showed that the differential bacteria in the CG all belonged to the phylum Firmicutes, while those in the EG all belonged to the phylum Bacteroidetes. As a natural antibiotic, the antibacterial effect of thymol is concentration-dependent. Research has found that adding plant essential oil containing 0.01 mg/mL thymol to microbial culture media resulted in inhibition rates of 29.63% and 22.22% against Rhizoctonia solani and strawberry root rot pathogen, respectively. As the concentration of thymol increased, its inhibition rates against both pathogens showed an upward trend, reaching 100% at a concentration of 0.5 mg/mL [33]. Additionally, different bacteria exhibit varying sensitivities to thymol. Studies indicate that Gram-positive bacteria are more sensitive to thymol compared to Gram-negative bacteria, and thymol primarily inhibits the growth of Gram-positive bacteria by interfering with cell membrane formation [34]. This may be because Gram-negative bacteria (such as Bacteroidetes) possess an outer membrane rich in lipopolysaccharides and other substances, which restricts the diffusion of hydrophobic compounds like thymol across the membrane. In contrast, Gram-positive bacteria (such as Firmicutes) lack an outer membrane and are only enveloped by a peptidoglycan wall, which is not dense enough to resist small antimicrobial molecules, making it easier for them to enter the cell membrane [35,36]. Therefore, thymol may inhibit the growth of Firmicutes by suppressing biofilm formation in these bacteria, thereby promoting the growth and reproduction of Bacteroidetes. Furthermore, in the correlation analysis between differential rumen bacteria and rumen fermentation parameters, Lachnospiraceae and Lachnospirales showed a positive correlation with butyrate content. Since Lachnospiraceae belongs to the order Clostridiales and is one of the main bacterial families producing butyrate in the rumen, a decrease in its relative abundance may lead to reduced butyrate content in the rumen [37], which is consistent with the results of this trial. Thus, thymol may reduce butyrate content in the rumen by decreasing the relative abundance of Lachnospiraceae. To further explore the potential influence of dietary thymol supplementation on the predicted functional profile of the rumen microbiota, Tax4Fun functional prediction followed by intergroup comparison was performed. The results showed that several predicted functional pathways, particularly those related to cellular processes and signaling and the biosynthesis of other secondary metabolites, exhibited nominal differences between the CG and EG based on raw *p*-values. However, these differences did not remain statistically significant after FDR correction. Therefore, these results should be interpreted as exploratory evidence suggesting possible functional responses of rumen microbial communities to dietary thymol supplementation. Because these pathways were inferred from 16S rRNA sequencing data, further validation using metagenomic, metatranscriptomic, metabolomic, or direct microbial metabolic analyses is required to confirm the actual functional changes and microbial metabolic activity.

## 5. Conclusions

Under the conditions of the present study, dietary supplementation with 30 mg/kg thymol improved growth performance in Texel × Hazak replacement ewes, as reflected by increased TWG and ADG and a decreased F/G ratio. Thymol supplementation was also associated with changes in selected plasma biochemical indices, steroid hormone concentrations, rumen fermentation parameters, and rumen microbial community composition. The 16S rRNA sequencing results further indicated that dietary thymol supplementation was associated with altered rumen microbial diversity and changes in the relative abundance of selected bacterial taxa.

Overall, dietary thymol supplementation may have potential value in modulating growth-related physiological status, rumen fermentation characteristics, and rumen microbial ecology in replacement ewes. However, nutrient digestibility, metagenomic function, microbial metabolic activity, estrous-cycle stage, and subsequent reproductive performance were not directly evaluated in this study. Therefore, further studies integrating nutrient digestibility measurements, enzyme activity assays, metagenomic or metabolomic analyses, and reproductive-performance evaluation are needed to clarify the mechanisms underlying the effects of thymol on growth performance and rumen microbial ecology in replacement ewes.

## Figures and Tables

**Table 1:** Diet Composition and Nutritional Levels (Dry Matter Basis).

Feed Composition	Content %	Nutritional Level ^2^	Content
Mountain grass	36.00	DM (%)	92.30
Alfalfa	24.00	OM (%)	93.08
Corn	17.50	DE (MJ/kg)	16.34
Soybean meal	7.50	CP (%)	13.64
Wheat middlings	5.60	EE (%)	2.92
Wheat bran	5.00	NDF (%)	43.69
Cottonseed meal	2.00	ADF (%)	30.66
Premix ^1^	2.00	Ca (%)	0.90
Baking soda	0.40	P (%)	0.49
In total	100.00		

^1^ The premix provides per kilogram of concentrate supplement: Iron 127.50 mg, Zinc 293.25 mg, Manganese 190.80 mg, Copper 13.05 mg, Selenium 1.18 mg; Vitamin A 16000 IU, Vitamin D_3_ 4080 IU, Vitamin E 82 mg, Niacinamide 32 mg, Biotin 0.08 mg. ^2^ Nutritional values are based onlaboratory analysis.

**Table 2:** Effects of Dietary Thymol Supplementation on the Body Weight of Replacement Ewes.

Items	CG	EG	SEM	*p*
Initial Weight kg	42.87	42.62	1.09	0.82
Final Weight kg	54.78	57.19	1.10	0.04
TWG kg	11.91	14.57	0.45	<0.001
ADG g	198.46	242.89	7.50	<0.001
Actual feed intake g	1295.33	1267.33	4.96	0.56
F/G	6.55	5.26	0.27	<0.01

Note: CG, control group; EG, experimental group supplemented with 30 mg/kg thymol in the diet. Actual feed intake was calculated as the amount of feed offered minus feed refusals and is presented on an as-fed basis. *n* = 15 per group. *p* < 0.05 indicates a significant difference, *p* < 0.01 indicates a highly significant difference, and *p* > 0.05 indicates no significant difference.

**Table 3:** Effect of Dietary Thymol Supplementation on Plasma Biochemical Indices in Ewes.

Items	CG	EG	SEM	*p*		
				TRT	Time	TRT × Time
TP (g/L)	60.21	63.19	0.71	<0.01	0.09	<0.0001
ALB (g/L)	22.02	23.39	0.31	<0.01	0.01	<0.0001
GLB (g/L)	38.20	39.79	0.63	0.08	0.56	<0.0001
CREA (mmol/L)	66.64	65.33	2.78	0.74	<0.0001	<0.01
UREA (mmol/L)	4.21	4.59	0.15	0.08	<0.0001	0.30
Glu (mmol/L)	3.55	4.07	0.10	<0.001	0.03	<0.0001
T-Bil (mmol/L)	0.61	0.56	0.03	0.21	<0.0001	0.49
D-Bil (mmol/L)	0.30	0.20	0.03	0.03	<0.0001	0.05
TC (mg/dL)	1.09	1.29	0.03	<0.0001	<0.0001	<0.0001
TG (mg/dL)	0.24	0.28	0.01	<0.01	<0.0001	<0.01
HDL-C (mg/dL)	0.90	0.87	0.02	0.38	<0.0001	0.05
LDL-C (mg/dL)	0.41	0.33	0.02	<0.001	0.09	0.17
AST (U/L)	122.36	105.82	2.82	<0.0001	<0.0001	0.03
ALT (U/L)	19.09	17.61	0.68	0.13	0.51	0.04
ALP (U/L)	231.34	243.62	9.27	0.35	0.02	<0.01
CK (U/L)	167.71	165.58	5.82	0.80	<0.0001	0.39
LDH (U/L)	441.62	445.93	7.72	0.69	<0.0001	<0.01
γ-GT (U/L)	61.65	48.73	1.64	<0.0001	0.12	0.14
AST/ALT	6.64	6.48	0.28	0.70	<0.001	0.78

Note: CG, control group; EG, experimental group supplemented with 30 mg/kg thymol in the diet. *n* = 15 per group. TRT represents the treatment effect, Time represents the time effect, and TRT × Time represents the treatment-by-time interaction effect. *p* < 0.05 indicates a significant difference, *p* < 0.01 indicates a highly significant difference, and *p* > 0.05 indicates no significant difference.

## Data Availability

The raw 16S rRNA gene sequencing data generated in this study have been deposited in the NCBI Sequence Read Archive. The data are publicly available under BioProject accession number PRJNA1490116. The original contributions presented in this study are included in the article/Supplementary Material. Further inquiries can be directed to the corresponding author.

## Supplementary Materials

The following supporting information can be downloaded at https://www.mdpi.com/article/10.3390/life16071162/s1, Table S1: Effects of Thymol Supplementation on the Body Weight of Replacement Ewes. Table S2: Effect of Dietary Thymol Supplementation on plasma Biochemical Indices in Ewes. Table S3: Effect of thymol on blood steroid hormone levels in ewes. Table S4: Effect of thymol on rumen fermentation parameters in ewes. Table S5: Effect of thymol on rumen microbial diversity in ewes.

## Abbreviations

The following abbreviations are used in this manuscript:

DM	Dry Matter
OM	Organic Matter
DE	Digestible Energy
CP	Crude Protein
EE	Ether Extract
NDF	Neutral Detergent Fiber
ADF	Acid Detergent Fiber
Ca	Calcium
P	Phosphorus
TP	Total Protein
ALB	Albumin
GLB	Globulin
CREA	Creatinine
UREA	Urea
Glu	Glucose
T-Bil	Total Bilirubin
D-Bil	Direct Bilirubin
TC	Total Cholesterol
TG	Triglycerides
HDL-C	High-Density Lipoprotein Cholesterol
LDL-C	Low-Density Lipoprotein Cholesterol
AST	Aspartate Aminotransferase
ALT	Alanine Aminotransferase
ALP	Alkaline Phosphatase
CK	Creatine Kinase
LDH	Lactate Dehydrogenase
γ-GT	y-Glutamyl Transferase
E2	Estradiol
E1	Estrone
A4	Androstenedione
DHEA	Dehydroepiandrosterone
Aldo	Aldosterone
P4	Progesterone
F	Cortisol
B	Corticosterone
